# Diagnostic Algorithm for Intracranial Lesions in the Emergency Department: Effectiveness of the Relative Brain Volume and Hounsfield Unit Value Measured by Perfusion Tomography

**DOI:** 10.7759/cureus.61591

**Published:** 2024-06-03

**Authors:** Juan Antonio Alvaro-Heredia, Luis A Rodríguez-Hernández, Héctor A Rodríguez-Rubio, Isidro Alvaro-Heredia, Michel G Mondragon-Soto, Ivan A Rodríguez-Hernández, Edgardo de Jesus Mateo-Nouel, Eliezer Villanueva-Castro, Rodrigo Uribe-Pacheco, Elvira Castro-Martinez, Guillermo A Gutierrez-Aceves, Sergio Moreno-Jiménez, Ignacio Reyes-Moreno, Alberto Gonzalez-Aguilar

**Affiliations:** 1 Neurological Surgery, National Institute of Neurology and Neurosurgery, Mexico City, MEX; 2 Spine Surgery, National Institute of Rehabilitation, Mexico City, MEX; 3 Emergency Medicine, National Institute of Neurology and Neurosurgery, Mexico City, MEX; 4 Radiosurgery, National Institute of Neurology and Neurosurgery, Mexico City, MEX; 5 Neurosurgery-Radiosurgery, The American British Cowdray (ABC) Medical Center, Mexico City, MEX; 6 Neuro-Oncology, The American British Cowdray (ABC) Medical Center, Mexico City, MEX

**Keywords:** lymphoma, glioblastoma, metastasis, abscesses, perfusion parameters, intracranial brain tumors, ct perfusion

## Abstract

Background

Early treatment of intracranial lesions in the emergency department is crucial, but it can be challenging to differentiate between them. This differentiation is essential because the treatment of each type of lesion is different. Cerebral computed tomography perfusion (CTP) imaging can help visualize the vascularity of brain lesions and provide absolute quantification of physiological parameters. Compared to magnetic resonance imaging, CTP has several advantages, such as simplicity, wide availability, and reproducibility.

Purpose

This study aimed to assess the effectiveness of Hounsfield units (HU) in measuring the density of hypercellular lesions and the ability of CTP to quantify hemodynamics in distinguishing intracranial space-occupying lesions.

Methods

A retrospective study was conducted from March 2016 to March 2022. All patients underwent CTP and CT scans, and relative cerebral blood volume (rCBV) and HU were obtained for intracranial lesions.

Results

We included a total of 244 patients in our study. This group consisted of 87 (35.7%) individuals with glioblastomas (GBs), 48 (19.7%) with primary central nervous system lymphoma (PCNSL), 45 (18.4%) with metastases (METs), and 64 (26.2) with abscesses. Our study showed that the HUs for METs were higher than those for GB (S 57.4% and E 88.5%). In addition, rCBV values for PCNSL and abscesses were lower than those for GB and METs. The HU in PCNSL was higher than those in abscesses (S 94.1% and E 96.6%).

Conclusion

PCT parameters provide valuable information for diagnosing brain lesions. A comprehensive assessment improves accuracy. Combining rCBV and HU enhances diagnostic accuracy, making it a valuable tool for distinguishing between lesions. PCT's widespread availability allows for the use of both anatomical and functional information with high spatial resolution for diagnosing and managing brain tumor patients.

## Introduction

It can be challenging to distinguish between intracranial lesions in an emergency department due to the urgency of early treatment. Oftentimes, the only available neuroimaging study is a cerebral computed tomography perfusion (CTP) scan, which sometimes cannot distinguish between the main types of lesions [1,2]. This is a problem because the treatment for each type of lesion is different, so it is essential to differentiate between them before surgery. Non-invasive in vivo methods are needed to assess and improve the accuracy of CTP diagnosis for intracranial lesions. Determining X-ray attenuation coefficients is one method that can help diagnose these lesions by differentiating abnormal tissues from normal ones using dose distribution and contrast differences [3-6]. Another method is perfusion imaging, which can be added to CTP to visualize the vascularity of brain lesions and quantify physiological parameters, such as blood flow, blood volume, mean transit time, and time to peak [7-9]. CTP has several advantages over magnetic resonance imaging, including simplicity, wide availability, and reproducibility. A study was conducted to evaluate the effectiveness of quantifying density by Hounsfield units (HU) in hypercellular lesions and quantifying hemodynamics by CTP in differentiating intracranial space-occupying lesions [10].

## Materials and methods

We reviewed and analyzed the clinical data and medical files of patients who had a confirmed histologic diagnosis by a neuropathologist from March 2016 to March 2022 at the National Institute of Neurology and Neurosurgery in Mexico City. Before any neurosurgical treatment, all patients underwent CT and CTP. The institutional review board approved this study, with approval number INNN 43-22.

Image acquisition

CTP was performed with a Sensation 16 (Siemens, Germany) multislice helical tomography (16 slices). Before the CTP study, a non-contrast CT scan of the skull was performed to locate the region of interest. During the CTP study, a non-ionic contrast medium (350 mg/100 mL) of 40 mL (iohexol) was injected through a 20 G caliber antecubital intravenous line using an automated injector at a flow rate of 5 mL/s. A cine series was acquired using the following parameters: 80 kV, 80 mA, and 1 second per rotation.

After administering the contrast medium, a five-second cine scanner was started five seconds later. The scanner then took one axial image every two seconds for the remaining 52 seconds, making the total acquisition time 57 seconds. Using CT Perfusion 3 software on the Advantage Windows workstation (version 4.2, GE Medical Systems, USA), perfusion maps of relative cerebral blood volume (rCBV) were generated for all patients. Regions of interest (ROIs) were manually placed in the tumor-enhancing area, peritumoral region, and contralateral normal-appearing white matter, as confirmed on both contrast-enhanced and non-contrast-enhanced CT images. The peritumoral region was defined as low density around the tumor, which was enhanced on the CT image. Parameters of CTP were normalized by those of the contralateral normal-appearing white matter to normalize parameters in the tumor (rCBV) and peritumoral region.

Statistical analysis

Demographics and different histopathological subgroups were analyzed using descriptive statistics. To determine the sensitivity and specificity parameters, an analysis was carried out, from which the positive predictive value and negative predictive value were obtained. This was done with a contingency table, which provided various probable results for the CT. Both CTP and CT were performed on all patients, and rCBV and HU were obtained for all intracranial lesions. These parameters were evaluated with the Mann-Whitney U test and receiver operating characteristics (ROC) analysis with an area under the curve (AUC) calculation to evaluate and compare prognostic performance. In addition, we compared these characteristics and obtained four groups: >30 HU/>2rCBV, >30 HU/<2rCBV, <30HU/>2 rCBV, and <30HU/<2rCBV. The results were analyzed using a binary logistic regression analysis model. The odds ratio (OR) and 95% confidence intervals (CIs) of each variable were estimated. Statistical significance was calculated using Pearson's chi-square and unpaired t-test with confidence levels of 95%. A P-value <0.05 was considered to be significant.

## Results

**Figure 1:**
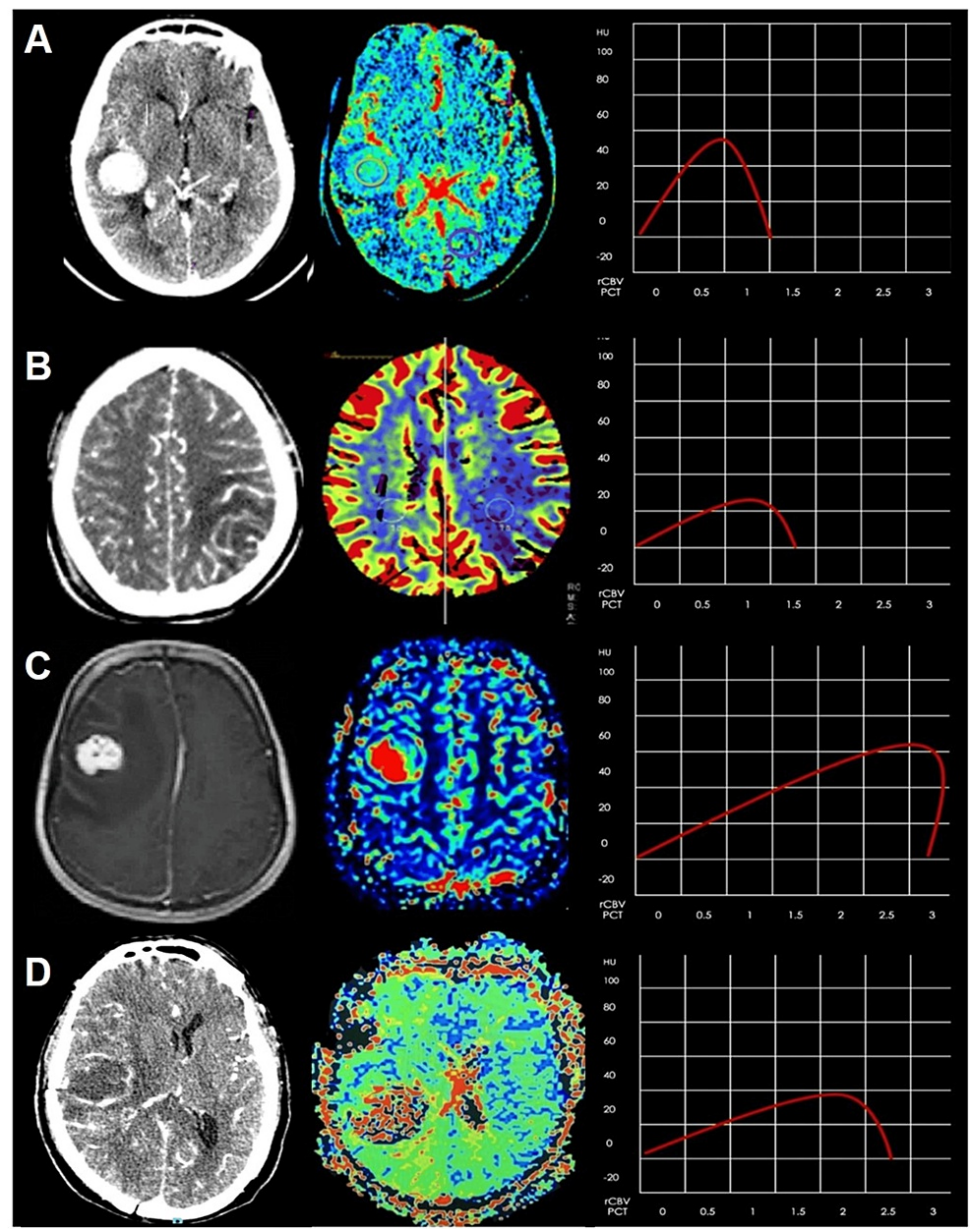
PCT/CT images, HU, and color maps in four different histologically proven lesions: (A) PCNSL, (B) abscess, (C) METs, and (D) GB. PCT/CT: perfusion computed tomography/computed tomography, PCNSL: primary central nervous system lymphoma, METs: metastases, GB: glioblastoma

This study was conducted retrospectively in a tertiary care hospital in Mexico City between March 2016 and March 2022. It included 244 patients, all of whom had a confirmed histologic diagnosis by a neuropathologist. The patients had a mean age of 54 years, with 99 (40.6%) women and 145 (59.4%) men. The study groups comprised 87 (35.7%) for glioblastomas (GBs), 48 (19.7%) for primary central nervous system CNS lymphoma, 45 (18.4) for metastases (METs), and 64 (26.2%) for abscess (Table 1, Figure 1).

Table 1 shows the demographic and clinical details. Figure 1 presents the PCT/CT images, HU, and color maps of four histologically proven lesions: CNS lymphoma, abscess, and METs.

**Table 1:** Demographics of the cases

Parameter	n(%)
Male	145 (59.4)
Female	99 (40.6)
Glioblastoma	87 (35.7)
Metastases	45 (18.4)
Lymphoma	48 (19.7)
Abscess	64 (26.2)

The rCBV maximum values were 4 for metastasis and 3.4 for PCNSL; the minimum was 0.2 for abscess and 0.6 for PCNSL. In the HU values, the maximum value was 55 for PCNSL and 43 for metastasis; the minimum was 12 for GB and 14 for abscesses. According to Kruskal-Wallis H results presented in Table 2, rCBV shows a significant difference according to the four intracranial lesions (X^2^ = 162.10; p ≤ 0.05) and for HU (X^2^ = 129.46; p ≤ 0.05) (Table 2).

**Table 2:** Statical values of rCBV and HU in different intracerebral lesions rCBV: relative cerebral blood volume; HU: Hounsfield unit; Asymp. Sig (Kolmogorov-Smirnov test), mean rank (Kruskal-Wallis H test)

Diagnosis		rCBV	HU
Glioblastoma	Median (std. deviation)	3.94 (+1.30)	20.89 (+5.73)
	Skew	1.58	1.54
	Kurtosis	2.61	3.37
	Asymp. sig	0.000	0.000
	Mean rank	192.24	73.34
Metastases	Median (std. deviation)	2.04 (+1.23)	31.80 (+4.46)
	Skew	0.00	-0.30
	Kurtosis	-1.60	4.14
	Asymp. sig	0.000	0.000
	Mean rank	118.17	173.34
Lymphoma	Median (std. deviation)	1.46 (+0.92)	34.08 (+5.43)
	Skew	0.96	0.49
	Kurtosis	-0.19	4.54
	Asymp. sig	0.001	0.000
	Mean rank	98.24	197.94
Abscess	Median (std. deviation)	0.68 (+0.34)	23.08 (+5.48)
	Skew	1.04	0.04
	Kurtosis	0.08	-0.40
	Asymp. sig	0.000	0.200
	Mean rank	48.95	96.99

ROC analysis for differentiating PCNSL, abscess, METs, and GB with rCBV showed a higher discriminatory value (AUC of 0.909, 95% CI 0.874-0.943, p = 0.000). The mean cut-off value was 2.08, with a sensitivity and specificity of 81.8% and 88.2%, respectively. ROC analysis was performed for differentiating the same intracerebral lesions with HU, showing a discriminant value (AUC of 0.885, 95% CI 0.835-0.935, p = 0.000). The mean cut-off value was 30.05 with a sensitivity and specificity of 85.8% and 79.2%, respectively.

Comparison of GBs and METs

The rCBV values of GB were found to be greater than those of METs, but the HU values were low. On the other hand, we observed that the HU of METs was greater than that of GB. We could identify metastases with sensitivity at 57.4% and specificity at 88.5% (Table 3).

Comparison of CNS lymphoma and abscess 

The study found that both PCNSL and abscess had lower rCBV values compared to GB and METs. In addition, the HU in PCNSL was higher than that of abscess, with sensitivity at 94.1% and specificity at 96.6% (Table 3).

Table 3 presents the positive and negative predictive values between different subgroups. 

**Table 3:** Sensitivity and specificity of the mean rCBV and HU to identify different intracerebral lesions and the positive and negative predictive values between them rCBV: relative cerebral blood volume; HU: Hounsfield unit; PPV: ‎positive predictive value; NPV: ‎negative predictive value

	Abscess (n = 64)	Glioblastoma (n = 87)	Lymphoma (n = 48)	Metastases (n = 45)
Sensitivity	84.4	81.4	94.1	57.4
Specificity	96.2	87.9	96.9	88.5
PPV	88.5	76.1	88.9	54
NPV	94.6	89	98.4	89.8

Variables that were independently associated have been studied with the four study groups: >30 HU/>2rCBV, >30 HU/<2rCBV, <30HU/>2 rCBV, and <30HU/<2rCBV (Table 4).

**Table 4:** Comparison and analysis of the HU and rCBV groups rCBV: relative cerebral blood volume; HU: Hounsfield unit; rCBV: relative cerebral blood volume, P: p-value

	rCBV		
	<2 n(%)	>2 n(%)	P-value
HU <30			
Glioblastoma	2(3)	80(95.2)	0.000
Metastases	4(6.1)	2(2.4)	0.406
CNS lymphoma	5(7.6)	2(2.4)	0.241
Abscess	55(83.3)	0(0.0)	0.000
HU >30			
Glioblastoma	0(0.0)	5(13.5)	0.017
Metastases	15(26.3)	24(64.9)	0.000
CNS lymphoma	33(57.9)	8(21.6)	0.001
Abscess	9(15.8)	0(0.0)	0.011

Those with significant differences (p < 0.05) were abscess on <30HU/<2rCBV (OR = 2.64; 95%CI = 1.46-14.90), CNS lymphoma on >30 HU/<2rCBV (OR = 4.91; 95%CI = 1.94-12.79), GB on >30 HU/>2rCBV (OR = 6.42; 95%CI = 1.13-16.58), and metastases on <30HU/>2 rCBV (OR = 5.16; 95%CI = 2.11-12.66) (Table 5).

**Table 5:** Result of the multivariable analysis rCBV: relative cerebral blood volume; HU: Hounsfield unit; P: p-value; OR: odds ratio; CI: confidence interval

	rCBV <2	P	OR	CI	rCBV >2	P	OR	CI
HU <30	Abscess	0.000	2.64	1.46-14.90	Glioblastoma	0.000	6.42	1.13-16.58
HU >30	CNS lymphoma	0.001	4.91	1.94-12.79	Metastases	0.000	5.16	2.11-12.66

**Figure 2:**
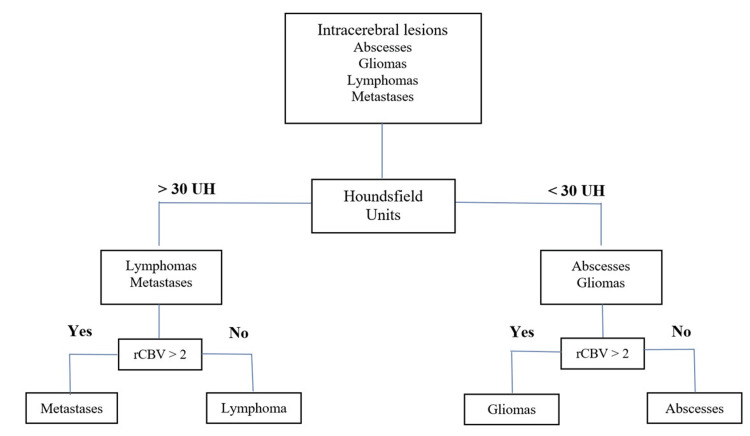
Flow diagram proposed in the present study (the patient on the top undergos several nodes to discriminate intracerebral lesions). rCBV: relative cerebral blood volume; HU: Hounsfield unit

Figure 2 presents a flow diagram that can help improve diagnostic accuracy.

## Discussion

This study aimed to examine CT and CTP parameters to distinguish between different conditions based on the cellularity and neovascularity of present lesions. The combined use of several parameters was found to be beneficial. We measured HU in all images by creating an ROI to establish the correlation between tumor cellularity and regional CTP values, and we found a strong correlation between the two. Advanced imaging techniques such as perfusion and spectroscopy are helpful in distinguishing between different types of lesions in the brain [11,12]. There have been few comprehensive studies using CTP to differentiate the rCBV of ring-enhancing intracranial lesions, although a few studies have been conducted to evaluate vascular permeability and perfusion in brain tumors and gliomas. CTP techniques have been validated and can be compared with xenon CT and MR perfusion techniques, showing consistent results [13-16]. 

Previously, the clinical efficacy of CTP in histologic grading of glioma has already been reported [16]. The differentiation between low-grade and high-grade gliomas was successfully achieved through perfusion studies. Low-grade tumors exhibited lower perfusion compared to high-grade tumors. Kamble RB et al. [17] found a statistically significant difference between the rCBV of low-grade glioma and high-grade glioma, with an rCBV cut-off value of 1.64 to differentiate between them. This value showed a sensitivity of 86.36% and a specificity of 100%. Similarly, Aronen et al. found that none of the low-grade gliomas had an rCBV greater than 1.5 [18].

However, there was no statistical difference in rCBV between GB and METs. Ellika SK et al. [19] demonstrated that the mean rCBV of high-grade gliomas is significantly higher, with a cut point for rCBV of >1.92, yielding 85.7% sensitivity and 100% specificity. Our study used CTP parameters to differentiate between GB, METs, PCNSL, and infective lesions. According to reports, the rCBV of GB obtained by CTP is more significant than those of PCNSL and abscess [19-21].

Infective lesions and primary CNS lymphoma show a reduced rCBV, as observed by Kamble RB et al. [17] in tuberculomas. To differentiate these lesions, we measured HU to determine cellularity. Our study shows a good correlation between cellularity and density with respect to HU, and we used a cut point for HU of 30, yielding 84.4% sensitivity, 96.2% specificity, and 94.1% and 96.9%, respectively. We found higher values of HU in PCNSL than infective lesions, and we concluded that a HU >30 and low rCBV values correlate with the lack of vascular proliferation and the higher cellularity of histopathological properties of PCNSL [10-13,22,23].

It should be noted that there is no standard threshold for rCBV to distinguish between different entities. However, we have adopted a cutoff value of 2 for our study. Our findings align with previous studies, such as those conducted by Okaii AL et al. [1], which also reported a good correlation with rCBV values >1.79.

Post-contrast acute kidney injury (AKI) and contrast-induced AKI can vary significantly depending on factors such as the presence or absence of risk factors, the volume and method of contrast material administration, and the specific patient population under examination [2]. In the absence of risk factors, the occurrence of contrast-induced AKI is very low. A major risk factor for kidney injury is a decline in kidney function, which may be due to chronic kidney disease or a current episode of AKI [23-26]. Other potential risk factors may include diabetes mellitus, heart failure, hypovolemia, proteinuria, and the use of intra-arterial (rather than intravenous) contrast material during medical procedures [27].

Limitations in the diagnosis accuracy of a single CTP parameter can be overcome by combining the analysis of rCBV and HU, which can be a useful tool in differentiating intracranial lesions. CTP techniques have been thoroughly validated and found to be comparable with xenon CT and MR perfusion techniques. These findings demonstrate a high level of agreement with quantitative results, highlighting the reliability and accuracy of CTP in assessing perfusion [24,27-32].

## Conclusions

The PCT parameters offer valuable information for preoperative diagnosis of PCNSL, abscesses, METs, and GB. We found that a comprehensive PCT parameter assessment improved diagnostic accuracy for intracranial lesions. This study showed that combining rCBV and HU can enhance diagnostic accuracy, making it a valuable tool for distinguishing between intracranial lesions. The widespread availability of PCT allows for the use of both anatomical and functional information with high spatial resolution for the initial diagnosis and subsequent management of brain tumor patients.

Our proposed imaging strategy has been highly accurate in differentiating intracranial lesions. When combined with the clinical context, our strategy is the best predictor for early and adequate treatment. This can be particularly beneficial for hospitals that perform neurological surgeries but lack access to reference MRI, which is a common issue in developing countries. Our treatment algorithm provides a standardized CTP approach, which is an effective alternative for diagnosing intracranial space-occupying lesions.
